# A case report of left ventricular lead implantation via total three-dimensional transseptal puncture after tricuspid valve replacement

**DOI:** 10.3389/fcvm.2023.1237967

**Published:** 2023-10-30

**Authors:** Jia Gao, Nan Zhang, Binghang Zhang, Meng Sun, Zhijun Meng, Min Guo, Rui Wang

**Affiliations:** ^1^ Department of Cardiology, First Hospital of Shanxi Medical University, Taiyuan, China; ^2^ Department of Clinical Laboratory, Shanxi Provincial People's Hospital of Shanxi Medical University, Taiyuan, China

**Keywords:** LV endocardial lead implantation, total 3-dimensional atrial septal puncture, CARTO 3 system, tricuspid valve replacement, pacemaker (PM)

## Abstract

**Background:**

Ventricular lead implantation is relatively difficult for patients with bradyarrhythmia after tricuspid valve replacement. Right atrial (RA) abnormalities often occurred in patients with tricuspid valve disease; conventional coronary sinus (CS) lead implantation is not easy to operate. Therefore, it is necessary to develop a safe method for implanting LV endocardial leads in patients after tricuspid valve replacement.

**Case presentation:**

A 76-year-old Asian woman who had been implanted with a metal tricuspid valve replacement 4 years ago was admitted to the Department of Cardiology for pacemaker implantation due to transient blackout related to persistent atrial fibrillation with long pauses. The patient's family rejected the surgical placement of an epicardial LV lead. Therefore, we first intended to operate LV lead implantation through the CS; however, the orifice of the CS was virtually difficult to seek. Ultimately, we utilized total 3-dimensional (T3D) transseptal puncture (TSP) under the guidance of the CARTO 3 system; thus, we implanted the LV endocardial lead, which contributed to the accurate puncture of the central fossa ovalis and ensured the safety of TSP in the case of RA enlargement. Meanwhile, the CARTO 3 system contributed to the localization of the LV lead to the LV free wall during implantation. All the intraoperative and postoperative pacemaker parameters were favorable; no intraoperative or postoperative complications occurred.

**Conclusions:**

This case report may provide a novel surgical approach for LV lead implantation in patients who underwent tricuspid valve replacement or patients who may benefit from cardiac resynchronization therapy but failed to implant CS lead.

## Introduction

In patients with bradyarrhythmia after metal tricuspid valve implantation, it is unconventional to perform routine right ventricular (RV) endocardial lead implantation; therefore, left ventricular (LV) lead implantation strategy must be adopted (1, 2). In clinical applications, LV lead implantation techniques include epicardial lead implantation via the coronary sinus (CS) or thoracotomy and endocardial lead implantation via atrial septal or ventricular septal puncture (3–5). LV epicardial lead implantation through the CS is the typical LV lead implantation method. However, conventional LV lead implantation through the CS cannot be achieved in 8%–10% of patients due to venous occlusion, nonviable coronary sinus anatomy, myocardial scar, or phrenic nerve stimulation (6). If we consider the difficulty and complexity of ventricular septal puncture and thoracotomy, atrial transseptal endocardial LV lead implantation, which is feasible and approximates a physiological state, is a favorable alternative (7). It was reported that traditional puncture via the femoral vein and modified puncture via the superior vena cava were alternatives to transseptal puncture (TSP) in LV lead implantation (8, 9). However, for patients who often exhibit RA-based structural abnormalities, such as expansion of volume, after tricuspid valve replacement, the aforementioned atrial septal puncture method may exhibit considerable difficulties and risks (10). Therefore, it is necessary to develop a safe and effective surgical approach of implanting LV lead for patients after tricuspid valve replacement. The patient consented to the publication of this case report and signed informed consent.

## Case presentation

A 76-year-old Asian woman was admitted to the Department of Cardiology owing to transient dizziness and blackouts, which had manifested for 1 year. The patient had undergone mechanical bileaflet heart valve (MTR29LFM, Sorin Biomedica Cardio S.P.A.) replacement 4 years ago owing to massive tricuspid regurgitation. Holter monitoring after admission exhibited persistent atrial fibrillation with long pauses (the longest interval was 4.976s with the symptom of dizziness); furthermore, the 24 h total heartbeat count was 72,767 beats, and the mean heart rate was 50 beats per minute. Transthoracic echocardiography (TTE) revealed that the metal tricuspid valve was hyperechoic and fixed in position, with no abnormal echo attachment, normal opening of the valve leaflets, as well as the enlargement of the right atrium (RA) (54*59 mm), left atrium (57 mm), and left ventricle (52 mm). The biochemical indicators (e.g., electrolyte and thyroid function) exhibited no significant abnormalities. In addition, the patient had consumed warfarin for a long duration after tricuspid valve replacement. No long-term medication history that affected heart rate was observed.

**Figure 1:**
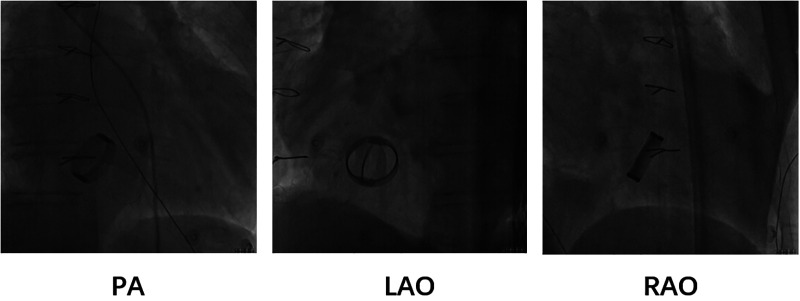
A preoperative chest radiograph of the patient.

**Figure 2:**
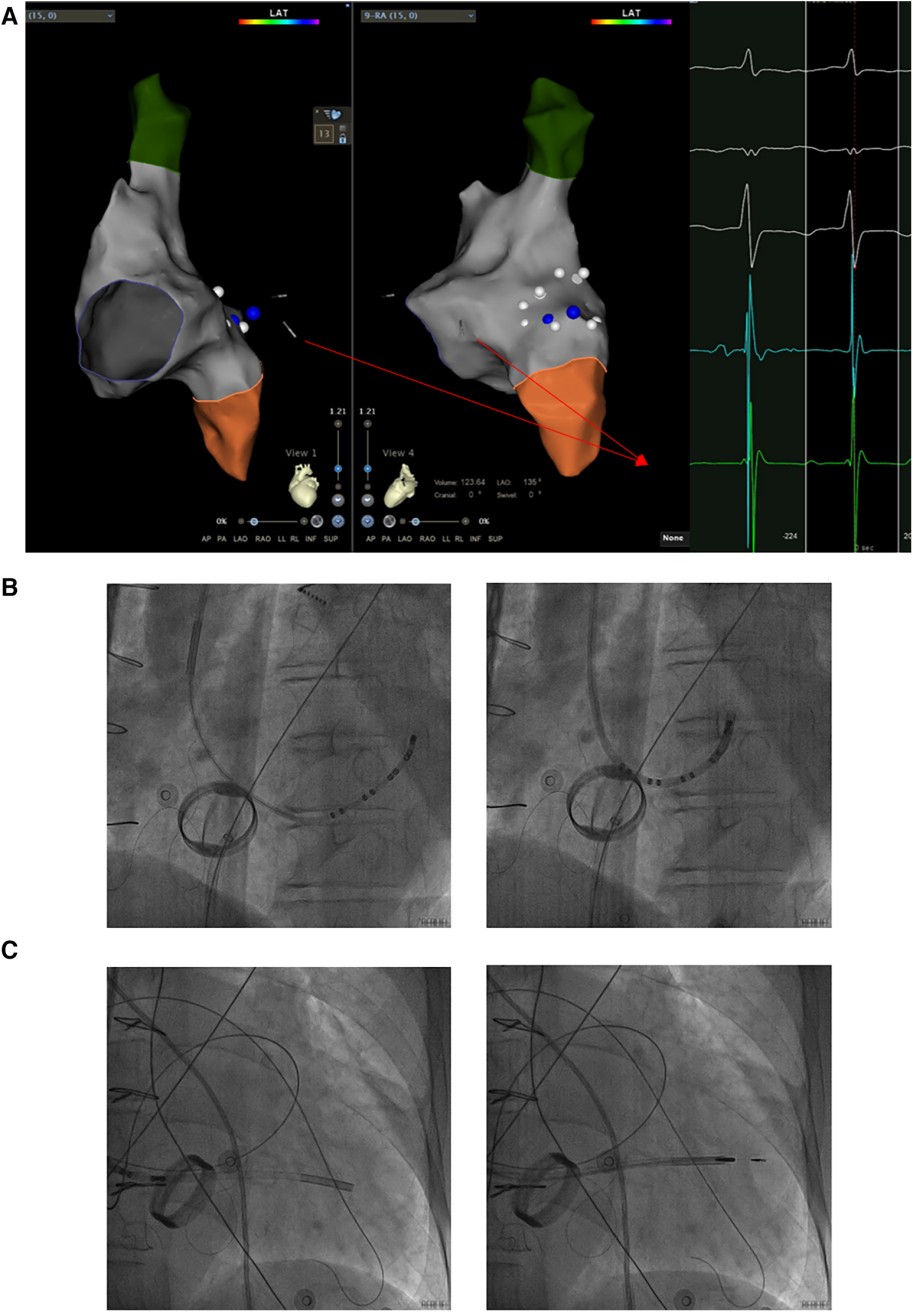
The LV lead was implanted into the LVFW along a sureValve 6250VIS under the guidance of CARTO 3 and x-ray imaging. (**A**) The CS lead was maneuvered into the LVFW through the mitral valve under the guidance of CARTO 3 and the potential of the CS lead. The potential of the CS lead pointing toward the LVFW is shown in the red box. (**B**) The CS lead which inserted into the Attain Command + SureValve 6250VIS passed through the centre of FO via the axillary vein followed by under 3D mapping and x-ray system. (**C**) Along the CS lead, SureValve 6250VIS was placed toward the LVFW. The LV lead was placed along the SureValve 6250VIS and rotated into LVFW. The red arrow shows the potential of the CS lead; the yellow box shows the potential at the center of the FO; the blue box shows the potential at the edge of the FO; LV: left ventricular; LVFW: left ventricular free wall; CS: coronary sinus; FO: fossa ovalis.

**Figure 3:**
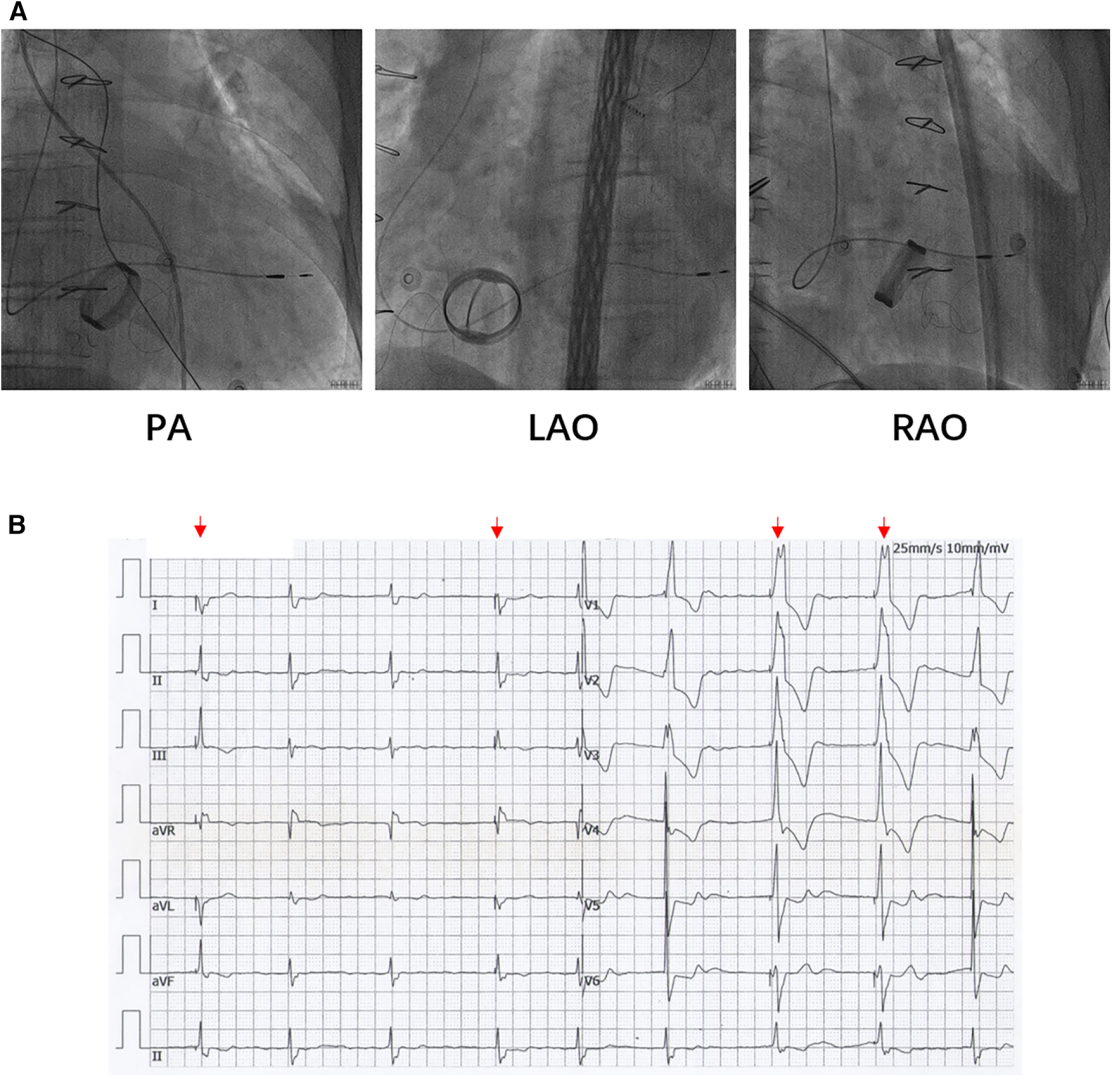
(**A**) The LV lead was placed to LVFW under x-ray guidance. (**B**) Postoperative ECG of patients. The red arrows indicate the pacing rhythm. ECG: electrocardiograph.

Based on the preceding results, the patient was diagnosed with transient dizziness and blackout related to long-term persistent atrial fibrillation with long pauses for which ventricular pacemaker implantation was required (I, C) according to the 2018 ACC/AHA/HRS guideline on the evaluation and management of patients with bradycardia and cardiac conduction delay. However, due to the tricuspid mechanical valve, it was impossible to implant lead in the right ventricle, which is the conventional practice. A preoperative chest radiograph of the patient was depicted in Figure 1. In addition, the patient's family rejected the surgical placement of an epicardial LV lead. Therefore, we first considered the implantation of LV lead through the CS; however, due to the large and abnormal RA, the surgeons could not locate the orifice of the CS after many intraoperative attempts. Meanwhile, to avoid the risk pertaining to the incarcerated metal valve during the searching, we decided to implant LV lead through TSP, after consultation with the patient's relatives. In consideration of the enlargement and variability of the RA, the risk pertaining to the incarcerated metal valve, and the safety of TSP, we decided to puncture the septum using the total three-dimensional (T3D) technique. T3D transseptal puncture based on the CARTO 3 system and the electrographic characteristics of fossa ovalis (FO) enables surgeons to puncture the center of the FO, and the technique has been expertly utilized in the radiofrequency ablation of cardiac tachyarrhythmia (11). We obtained the femoral venous access, and we utilized the ablation catheter (ThermoCool SmartTouch, Biosense Webster, Diamond Bar, California) to create the 3D shell of the RA as well as the superior vena cava and inferior vena cava under the CARTO3 system (Boisense Webster, Diamond Bar, CA, USA). The ablation catheter was sent to the 4 to 5 o'clock position of the superior vena cava; subsequently, it was slowly pulled down at left anterior oblique (LAO) 135° and LAO 45° projections. During the process of descending, when the “jump” appeared and atrial signals decreased to less than 1/3 of the atrial signals, we initially labeled FO and repeated the aforementioned operation 3 times. Based on those points, we moved the catheter up and down and left and right; furthermore, we noted the atrial signals and determined the centre (blue point) of the FO (Figure 2A). The electrical amplitude at the central FO was significantly lower than the FO border (Figure 2A). The puncture needle could be recognized as a bipolar catheter when its tail was connected to the CARTO by a bipolar clip, and its tip or electric potential became visible during 3D modeling. Under the guidance of CARTO 3, we successfully punctured the center of the FO; the outer sheath was utilized to repeatedly expand the puncture site, and a long guide wire was inserted into the LA as a guideline. The CS lead was adjusted to pass through the FO and mitral valve via the axillary vein, subsequently, maneuvered into the left ventricular free wall (LVFW) through the Tear-away Introducer System (HLS2509M, Merit Medical Systems, Inc., South Jordan, USA) followed by the Left-heart Delivery System (Attain Command + SureValve 6250VIS, Medtronic, Inc. Minneapolis USA) under 3D mapping and an x-ray system (Figure 2B). Along the CS lead, a SureValve 6250VIS was placed in the LA and pointed toward the LVFW. The LV lead (3,830, Medtronic, Inc. Minneapolis USA) was placed along the SureValve 6250VIS and rotated into the myocardium (Figure 2C). The parameters were as follows: ≥5.6 mV R-wave sensing, ≥10.3 mV current of injury (COI), 0.5 V pacing threshold, and 704 *Ω* impedance. Finally, the SureValve 6,250 VIS was torn off, the lead was connected to pacemaker, and the wound was stitched. x-ray images were illustrated in Figure 3A. The postoperative pacing parameters did not change significantly; there were no intraoperative or postoperative complications. Postoperative ECG was depicted in Figure 3B.

## Discussion

In this report, we first utilized T3D atrial septal puncture to implant an LV endocardial lead in a female patient after tricuspid valve replacement; thus, the safety of atrial septal puncture in the RA enlargement case was ensured. This study provides a novel procedure for LV lead implantation in patients after tricuspid valve replacement, and in patients who can potentially benefit from cardiac resynchronization therapy but failed to implant CS lead.

In traditional LV lead implantation, implantation through the CS is the most convenient and safest method. However, in patients who have undergone tricuspid valve replacement, due to RA enlargement and variation, the CS ostium was not easy to search out during the operation. Meanwhile, during the searching of the CS ostium, the metal valve may be incarcerated by the CS electrode which may lead to the malfunction of the tricuspid valve. The surgical approach that entails implanting an epicardial lead represents an alternative approach. Although the surgical approach exhibits a series of advantages (e.g., a lower risk of lead dislodgement, phrenic nerve stimulation, and robustness against CS anatomy-based limitations), the approach exhibits several crucial disadvantages: it is more invasive and traumatic and limited by adhesions and epicardial fat, exhibits prolonged postoperative recovery time, and increases the transmural dispersion of repolarization and delay conduction (12, 13). Therefore, even the surgical approach remains the main second-line option, and many patients reject the surgical approach in clinical practice. Therefore, it is necessary to develop an acceptable and effective surgical approach to implant LV lead for patients after tricuspid valve replacement.

Since Jais et al. reported the first placement of an LV endocardial pacing lead via the TSP in 1,998 (7); the procedure has been gradually adopted in clinical practice. Due to direct contact with endocardial tissue, compared with traditional LV epicardial pacing and cardiac resynchronization therapy (CRT), LV endocardial pacing displayed the following advantages: a lower threshold, lower diaphragm irritation, a wider selection of pacing sites, lower arrhythmia risk, and a more optimal approximation of the physiological state (14, 15). However, this technique has been utilized for LV pacing in a small number of patients for whom LV epicardial pacing has failed during CRT, partially owing to the following reasons: (1) the risk of lead thrombosis and embolism; (2) the difficulty of trans-atrial septal puncture guidance; and (3) the influence of the lead on the mitral valve.

In this report, T3D atrial septal puncture was utilized for the first time; thus, the implantation of a lead for LV endocardial pacing was facilitated. Based on the electrographic characteristics of the FO, T3D atrial septal puncture accurately punctured the central part of the atrial septum, which ensured that the long sheath could easily pass through the site of the atrial septal puncture and protected the safety of the lead after implantation. Meanwhile, according to the 3D reconstitution model of the RA, it could pass through the septal puncture site via the superior vena cava with relatively more ease which reduced the use of snare. In addition, SureValve 6250VIS was transited through the mitral valve and safely directed to the LVFW guided by the 3D mapping model and the tip potential of the CS lead.

## Conclusions

In conclusion, this trial indicated the usability and reliability of T3D atrial septal puncture in the implantation of LV endocardial leads and provided a novel approach for future implantation of LV lead in patients after tricuspid valve replacement, and in patients who may benefit from cardiac resynchronization therapy but failed to implant CS lead.

## Data Availability

The original contributions presented in the study are included in the article/Supplementary Material, further inquiries can be directed to the corresponding authors.
